# P2 Receptors and Platelet Activation

**DOI:** 10.1100/tsw.2002.106

**Published:** 2002-02-13

**Authors:** Satva P. Kunapuli

**Affiliations:** Department of Physiology, and the Sol Sherry Thrombosis Research Center, Temple University Medical School, Philadelphia, PA 19140, USA

**Keywords:** platelets, P2X1 receptor, P2Y1 receptor, P2T receptor, P2Y12 receptor, ADP receptor, platelet aggregation

## Abstract

Adenosine diphosphate (ADP) plays a crucial role in hemostasis and thrombosis by activating platelets. In platelets, the classical P2T receptor is now resolved into three P2 receptor subtypes: the P2Y1, the P2Y12, and the P2X1 receptors. Both pharmacological and molecular biological approaches have confirmed the role of the P2Y1 and P2Y12 receptors in the ADP-induced platelet fibrinogen receptor activation. The P2Y1 and the P2X1 receptors independently contribute to platelet shape change. Whereas the P2Y12 receptor mediates the potentiation of dense granule release reaction, both the P2Y1 and P2Y12 receptors play an important role in the ADP-induced phospholipase A2 activation. The signaling events downstream of these receptors leading to the physiological effects remain elusive, and they are yet to be delineated.

## INTRODUCTION

Platelets aggregate at the site of vascular damage to form a hemostatic plug. Abnormal activation of platelets leads to thrombosis, which in turn leads to stroke and myocardial infarction[1]. Adhesion of platelets to subendothelium results in the release, generation, or exposure of agonists that can activate platelets in a positive-feedback loop. Among these agonists are collagen (exposed), thrombin and thromboxane $A_2$ (generated), and ADP (adenosine diphosphate), epinephrine, and serotonin (released)[2]. ADP was identified as a platelet agonist[3], which is released upon activation of platelets along with ATP (adenosine triphosphate) and serotonin[4]. The importance of ADP in platelet activation is substantiated by bleeding diathesis in patients with deficiencies in storage of ADP, in mechanisms of secretion, or in ADP receptors[5,6,7,8,9].

## NOMENCLATURE OF P2 RECEPTORS

Receptors for nucleotides, designated P2 receptors, are divided into two main classes: ligand-gated ion channels (P2X) and G protein-coupled receptors (P2Y)[10]. Initially, all the physiological and intracellular signaling events triggered by ADP in platelets were attributed to a single cell surface receptor, designated P2T (thrombocyte P2 receptors)[11]. The historic studies and theories on the nature of the P2T receptor have been dealt in recent review articles[12,13,14].

## ADP RECEPTOR-COUPLED EFFECTOR SYSTEMS IN PLATELETS

ADP has several effects on the receptor-coupled effector systems in platelets:

- ADP regulates several second messenger systems in platelets by acting on cell surface P2 receptors[12,13,14,15].
- ADP causes rapid calcium influx into platelets in the presence of physiological extracellular calcium ion concentrations[16,17].
- ADP activates platelet phospholipase C (PLC), resulting in inositol 1,4,5-trisphosphate formation that leads to mobilization of calcium from intracellular stores[18,19,20].
- ADP inhibits stimulated platelet adenylyl cyclase through coupling to Gαi2 protein[21] and thereby decreases intracellular cAMP levels[22].
- ADP also activates phospholipase $A_2$ ($PLA_2$) and liberates arachidonic acid from membrane phospholipids that is converted to thromboxane $A_2$ [23].

## PHYSIOLOGICAL EFFECTS OF ADP ON PLATELETS

Activation of platelets by ADP results in shape change when discoid-shaped resting cells are rapidly converted to spiculated spheres[24], platelet aggregation, and granule secretion[4,24]. ADP-induced dense granule release is abolished by aspirin treatment indicating that arachidonic acid products generated through cyclo-oxygenase pathway are essential for ADP ability to cause secretion[4,23]. Several investigators reported that ADP causes release of alpha granule contents[12,25,26], but other laboratories have shown that ADP fails to expose P-selectin[27] or β-thromboglobulin[28] in aspirin-treated and washed platelets, indicating that this event may also require thromboxane $A_2$ generation.

## A MODEL FOR ADP-INDUCED PLATELET ACTIVATION

Although P2T was thought to be the single ADP receptor mediating all the physiological and intracellular signaling events triggered by ADP in platelets[11], we have resolved the P2T receptor into three P2 receptor subtypes. We used AR-C 66096, a potent antagonist of ADP-induced platelet aggregation, and α,β-meATP, a P2X1 receptor agonist, to distinguish the ADP-induced intracellular events[20]. AR-C 66096 failed to inhibit ADP-mediated intracellular calcium increases, inositol trisphosphate formation, or shape change, although it blocked ADP-induced inhibition of adenylyl cyclase. α,β-Methylene ATP (α,β-MeATP) causes rapid calcium influx[17], but it neither caused the inositol trisphosphate formation nor inhibited adenylyl cyclase[20]. Based on these observations we proposed the presence of three distinct P2 receptor subtypes on platelets[20]: one coupled to inhibition of adenylyl cyclase, designated $P2T_{AC}$ receptor; the second coupled to mobilization of calcium from intracellular stores through activation of PLC and inositol trisphosphate formation, designated $P2T_{PLC}$; and the third an ionotropic P2X1 receptor coupled to rapid calcium influx. Several other studies[29,30,31,32]

independently confirmed the three-receptor model by pharmacological approaches. The P2$T_{AC}$ receptor is also called P2cyc[31], P2$Y_{ADP}$[32], P2$Y_{AC}$[30,33], P2Y [34], and P2T[29].

We have isolated a cDNA clone encoding the P2Y1 receptor from a human platelet cDNA library and demonstrated that the P2Y1 receptor is the P2$T_{PLC}$, using the P2Y1 receptor selective antagonists[35]: adenosine-3'-phosphate-5'-phosphosulfate (A3P5PS); adenosine-3'-phosphate-5'-phosphate (A3P5P); and adenosine-2'-phosphate-5'-phosphate (A2P5P)[36]. The P2$T_{AC}$ receptor was cloned by four separate groups and designated P2Y12 receptor[37,38,39,40]. Unpublished reports from our laboratory and a recent study[37] have shown that P2Y12 receptor is antagonized by the AR-C series of compounds. Thus the concept of P2T receptor[11] is resolved into three P2 receptor subtypes, viz. P2Y1, P2Y12, and P2X1 receptors, each with distinct functions. Furthermore, several recent independent studies also support the three-receptor model by gene disruption approaches[33,41,42].

## LIGANDS OF PLATELET P2 RECEPTOR SUBTYPES

Selective agonists and antagonists can delineate the function of the platelet P2 receptor subtypes in platelets. α,β-MeATP was identified as a selective agonist on ligand gated P2X1 channels on platelets, leading to rapid influx of calcium[17,20,36]. Although ADP was believed to be an agonist at the P2X1 receptors, recent work from Mahout-Smith and co-workers has identified ATP, but not ADP, as the true agonist at the P2X1 receptors[43]. The cyclic pyridoxine-alpha-4,5-monophosphate (MRS 2219), was found to be a selective agonist at rat P2X1 receptors while the corresponding 6-azophenyl-2',5'-disulfonate derivative (MRS 2220) was a selective antagonist[44]. A number of adenosine bis-phosphates (viz., A3P5PS, A3P5P, A2P5P, MRS 2179) have been developed as the selective competitive antagonists of the P2Y1 receptor[35,45,46], and these compounds have been shown to selectively act at the platelet P2Y1 receptor without any effect on P2Y12 receptors[36,37,38,40]. Hydrolysis-resistant derivatives of 2-substituted ATP (e.g., AR-C 66096 and AR-C69931MX) have been developed as a potent inhibitors of ADP-induced platelet aggregation[47] and have been shown to selectively antagonize the P2Y12 receptor subtype when used at limited concentrations[20,36,37,48]. The thienopyridine derivatives, ticlopidine and clopidogrel, when administered *in vivo*, selectively abrogate ADP-induced inhibition of adenylyl cyclase and platelet aggregation[49,50,51] indicating that an active metabolite acts at the P2Y12 receptor but not at the P2Y1 receptor[38,52,53]. 2-Methylthio-AMP (2MeSAMP) is identified as a selective antagonist of the P2Y12 receptor[32,40]. In addition, benzoyl ATP is a nonselective antagonist at both the P2Y1 and P2Y12 receptors[54]. Both the P2Y1 receptor and the P2Y12 receptor are activated by ADP and 2-methylthio-ADP (2MeSADP), although with different potencies[12,13]. The P2Y1 receptor is the high affinity receptor for ADP, whereas 2MeSADP is at least 100-fold more potent at the P2Y12 receptor than at P2Y1 receptor[12,37,40]. The effects of these ligands on platelet P2 receptor subtypes are summarized in Table 1.

## ADP-INDUCED PLATELET SHAPE CHANGE

P2Y1 receptor selective antagonists[35] — A3P5PS, A3P5P, and A2P5P — inhibit ADP- or 2MeSADP-induced intracellular calcium mobilization and shape change in platelets[36]. The $EC_{50}$ for ADP at the cloned P2Y1 receptor is ~0.3 μM[55], which is also the dose sufficient for platelet shape change[24]. Furthermore, ADP fails to cause shape change in platelets from mice lacking $G_q$, indicating that signaling through $G_q$ is essential for ADP-induced shape change[56]. All the agents that cause platelet shape change, such as thrombin, thromboxane, and serotonin, also activate PLC[2]. Hence, PLC activation is the essential step in platelet shape change.

**TABLE 1**
**Platelet P2 Receptor Ligands**

| Compound (Reference) | P2Y12 Receptor | P2Y1 Receptor | P2X1 Receptor |
|---|---|---|---|
| *ADP[12,13,43]* | Agonist | Agonist | No effect |
| 2MeSADP[12,13] | Agonist | Agonist | ? |
| 2MeSAMP[32,40] | Antagonist | No effect | ? |
| α,β-MeATP[17,20,36] | No effect | No effect | Agonist |
| ATP[12,13,43] | Antagonist | Antagonist | Agonist |
| AR-C compounds[20,36,37,47,48] | Antagonist | No effect | No effect |
| A3P5PS, A3P5P, or A2P5P[35,36,37,38,40] | No effect | Antagonist | ? |
| MRS 2179[45,46] | No effect | Antagonist | No effect |
| MRS 2219[44] | No effect | No effect | Agonist |
| MRS 2220[44] | No effect | No effect | Antagonist |
| Benzoyl ATP[54] | Antagonist | Antagonist | ? |
| Active metabolite of clopidogrel[38,52,53] | Antagonist | No effect | ? |

Thus the P2Y1 receptor solely mediates ADP-induced platelet shape change through coupling to $G_q$ and subsequent activation of PLC. When intracellular calcium increases were blunted in platelets using an intracellular calcium chelator, however, ADP still caused shape change[57]. This calcium-insensitive shape change was blocked by selective inhibitors of p160ROCK, a Rho kinase downstream of RhoA[57]. P2Y1 receptor stimulation leads to activation of RhoA-Rho kinase pathway and PLC pathway, which independently contributes to platelet shape change. The signal transduction events downstream of the P2Y1 receptor contributing to ADP-induced platelet shape change have been recently discussed[57,58,59].

AR-C 66096, a selective antagonist of the P2Y12 receptor, did not inhibit ADP-induced shape change[20], indicating that the P2Y12 receptor does not play any significant role in shape change induced by ADP.

Although it was long believed that the P2X1 receptor does not contribute to platelet shape change, recent reports demonstrate that P2X1-evoked calcium influx not only potentiates P2Y1-mediated calcium responses[60], but also independently causes platelet shape change[61] (Fig. 1).

## ADP-INDUCED PLATELET AGGREGATION

The P2Y12 receptor is essential for ADP-induced platelet aggregation. A significant correlation was found between antagonist affinity constant values for eight nucleotide analogs as blockers of ADP-induced aggregation and adenylyl cyclase inhibition[62]. Selective antagonists of the P2Y12 receptor — ATP, AR-C 66096, and 2MeSAMP — block both ADP-induced adenylyl cyclase inhibition[20,32,63] and platelet fibrinogen receptor activation[47,63]. *In vivo* administration of ticlopidine and clopidogrel results in complete inhibition of both ADP-induced inhibition of adenylyl cyclase and aggregation[51]. Two patients with defective ADP-induced platelet adenylyl cyclase inhibition also had abnormal aggregation suggesting that the receptor coupled to inhibition of adenylyl cyclase is essential for platelet aggregation[8,9]. Finally, the platelets from mice deficient in the P2Y12 receptor do not aggregate upon treatment with ADP[42]. Hence the P2Y12 receptor activation is required for ADP-induced platelet aggregation.

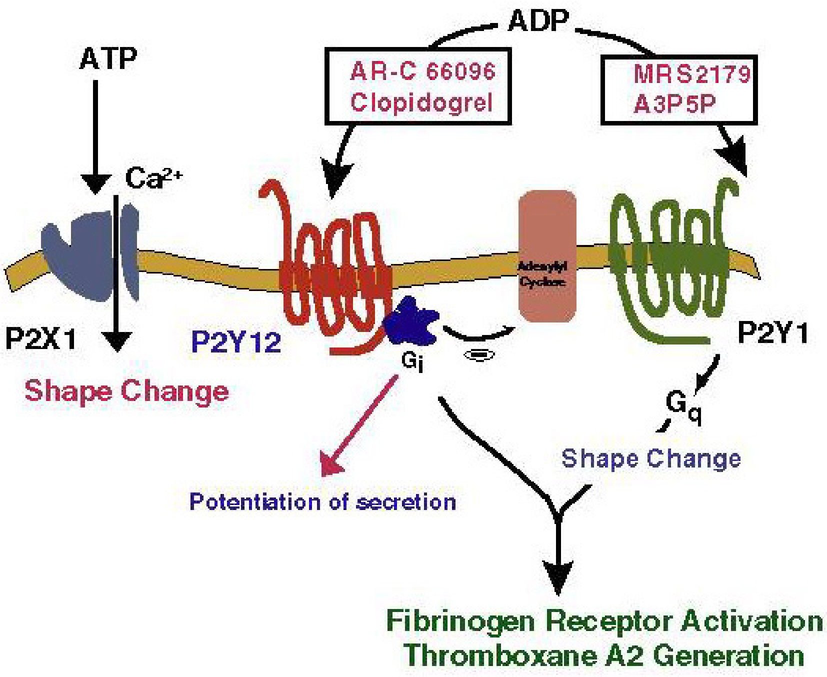

**FIGURE 1**. Role of P2 receptor subtypes in platelet activation

The P2Y1 receptor selective antagonists — A3P5PS, A3P5P, and A2P5P — also inhibit ADP-induced human[64] and mouse[65] platelet aggregation without blocking ADP-induced inhibition of adenylyl cyclase. Platelets from mice lacking the P2Y1 receptor or $G\alpha_q$ failed to mobilize calcium from intracellular stores, change shape, or aggregate in response to ADP[33,41,56]. Hence, intracellular signaling events from both the P2Y12 and P2Y1 receptors are essential for ADP-induced platelet aggregation. Inhibition of signaling through either receptor, by specific antagonists or receptor knock-outs, is sufficient to block ADP-induced platelet fibrinogen receptor activation. The P2Y1 receptor presumably couples to $G_q$ and causes intracellular calcium mobilization through the inositol trisphosphate pathway. In the presence of AR-C 66096, signaling through the P2Y12 receptor can be substituted by epinephrine acting on $\alpha_{2A}$ adrenergic receptors, coupled to $G_z$[64,66]. On the other hand, activation of serotonin receptors can replace signaling through the P2Y1 receptor in human[64], rabbit[67], or mouse[41] platelets through activation of $G_q$. Moreover, this novel mechanism of ADP-induced platelet aggregation can be mimicked by coactivation of two receptors coupled to $G_i$ and $G_q$, $\alpha_{2A}$ adrenergic receptors and serotonin receptors, respectively[64].

Interestingly, thromboxane $A_2$- or plasmin-induced platelet aggregation depends on costimulation of $G_i$ pathways by the secreted ADP through the P2Y12 receptor[68,69]. Thus, ADP-induced platelet aggregation results from concomitant signaling from both the P2Y12 and P2Y1 receptors (Fig. 1), a novel mechanism by which G protein-coupled receptors elicit a physiological response[64].

P2X1 receptor activation causes rapid calcium influx in the presence of extracellular calcium, but this event does not cause platelet aggregation or modulate ADP-induced platelet aggregation[64,70]. Furthermore, selective coactivation of the P2X1 receptors and either the P2Y12 or P2Y1 receptors also does not cause platelet aggregation[64], although P2X1 receptor potentiates P2Y1 receptor-mediated intracellular calcium increases[60]. Thus the P2X1 receptor-mediated rapid calcium influx causes platelet shape change, but it does not play any significant role in ADP-induced platelet aggregation.

These studies point to pitfalls in using receptor antagonists to delineate the role of the receptor in physiological function. The studies on ADP-induced platelet aggregation suggest that some agonist-induced physiological responses may require simultaneous activation of multiple receptor subtypes by the same agonist, resulting in converging signal transduction pathways leading to a physiological response. Thus, the role of another receptor subtype in an agonist-induced physiological event cannot be excluded in the studies with receptor specific antagonists.

## ADP-INDUCED THROMBOXANE A2 GENERATION

ADP-induced dense granule release depends on generation of thromboxane $A_2$, which causes release reaction by activating TP receptors. AR-C67085, a P2Y12 receptor selective antagonist, and A2P5P, a P2Y1 receptor selective antagonist, inhibited ADP-induced phospholipase $A_2$ activation and thromboxane $A_2$ generation, indicating that coactivation of the P2Y12 and P2Y1 receptors is essential for these events[71]. ADP does not cause thromboxane $A_2$ production in unstirred suspensions of platelets[28], which do not aggregate. It was argued that close cell-cell contact mediated by fibrinogen cross-linking is essential for ADP-induced thromboxane $A_2$ production. Snake venom–derived proteins applaggin, echistatin, and trigramin, which block fibrinogen binding to its receptor, have been shown to block ADP-induced platelet aggregation and thromboxane production[29,30]. SC49992, a fibrinogen receptor antagonist, also blocked ADP-induced platelet aggregation, phospholipase $A_2$ activation, and thromboxane $A_2$ production[71]. Whereas SC49992 blocked arachidonic acid–induced platelet aggregation, it failed to inhibit conversion of arachidonic acid to thromboxane $A_2$. Hence, ADP-induced arachidonic acid liberation, but not subsequent conversion to thromboxane $A_2$, requires outside-in signaling through the fibrinogen receptor. Induction of aggregation by the Fab fragment of LIBS6 antibody, which induces a fibrinogen-binding site on the integrin αIIbβ3, caused thromboxane $A_2$ generation[71].

In the presence of P2 receptor antagonists A2P5P or AR-C67085, however, LIBS6 failed to generate thromboxane $A_2$, suggesting that inside-out signaling through ADP receptors is also necessary for phospholipase $A_2$ activation[71]. Thus, inside-out signaling from both the P2Y1 and P2Y12 receptors is essential for phospholipase $A_2$ activation, resulting in arachidonic acid liberation and thromboxane $A_2$ generation (Fig. 1).

## DENSE GRANULE RELEASE REACTION

Given that thromboxane $A_2$ generated by ADP mediates dense granule release, what is the contribution of these P2 receptor subtypes to dense granule release? Platelet secretion induced by the thromboxane $A_2$ mimetic U46619 was unaffected by A3P5P, but P2Y12 receptor antagonists (such as AR-C66096 and AR-C69931-MX) inhibited U46619-induced platelet secretion, indicating an important role for $G_i$ signaling in platelet secretion[72,73]. Similarly, dense granule release reaction was abnormal in platelets from patients defective in the P2Y12 receptor[74]. Selective activation of either the P2Y12 receptor or the $\alpha_{2A}$ adrenergic receptor did not cause platelet secretion, but it potentiated U46619-induced platelet secretion[72].

Since $G_i$ signaling results in reduction of basal cAMP levels through inhibition of adenylyl cyclase, we investigated whether this signaling event potentiates platelet secretion. SQ22536 or dideoxyadenosine, inhibitors of adenylyl cyclase, failed to potentiate U46619-induced primary platelet secretion, which indicated that reduction in cAMP levels does not directly contribute to platelet secretion[72]. Thus signaling through the P2Y12 receptor by secreted ADP causes positive feedback on platelet secretion (Fig. 1). Furthermore, some patients with the common, ill-defined diagnosis of primary secretion defect could actually be heterozygous for the P2Y12 receptor defect[74].

## CONCLUSIONS AND FUTURE DIRECTIONS

Molecular mechanisms of ADP-induced platelet activation are becoming clear only now. First the resolution of the concept of P2T receptor into three components — P2Y1, P2Y12, and P2X1 receptors — helped to explain the intracellular and physiological effects of ADP on platelets. The interaction of signaling events downstream of the P2Y1 and P2Y12 receptors is a novel mechanism of physiological response and may indeed be a general mechanism of αIIbβ3 integrin activation by all physiological agonists. We speculate that the integrin activation on other cells also requires similar signaling mechanisms and this aspect remains to be established. Interestingly, mouse platelets deficient in the P2Y1 receptor can undergo partial aggregation with high concentrations of ADP[41]. The implications for this observation can range from a fourth P2 receptor subtype on platelets to the P2Y12 receptor coupling to other G proteins. The signaling mechanisms and cascades mediated by these three P2 receptor subtypes will provide a better understanding of ADP-mediated physiological responses in platelets and, generally, the molecular mechanisms of agonist-induced platelet activation.

## ACKNOWLEDGEMENTS

This work was supported by research grants HL60683, HL64943, and HL63933 from the National Heart Lung and Blood Institute, National Institutes of Health. It was performed during the tenure of an Established Investigator Award in Thrombosis from the American Heart Association and Genentech.

## References

1. Packham, M.A. (1994) Role of platelets in thrombosis and hemostasis. *Can. J. Physiol. Pharmacol.* **72**, 278–284.
2. Hourani, S.M.O. and Cusack, N.J. (1991) Pharmacological receptors on blood platelets. *Pharmacol. Rev.* **43**, 243–298.
3. Gaarder, A., Jonsen, A., Laland, S., Hellem, A.J., and Owren, P. (1961) Adenosine diphosphate in red cells as a factor in the adhesiveness of human blood platelets. *Nature* **192**, 531–532.
4. Mills, D.C.B., Robb, I.A., and Roberts, G.C.K. (1968) The release of nucleotides, 5-hydroxytryptamine and enzymes from human blood platelets during aggregation. *J. Physiol.* **195**, 715–729.
5. Holmsen, H. and Weiss, H.J. (1970) Hereditary defect in the platelet release reaction caused by a deficiency in the storage pool of platelet adenine nucleotides. *Br. J. Haematol.* **19**, 643–649.
6. Holmsen, H. and Weiss, H.J. (1979) Secretable storage pools in platelets. *Annu. Rev. Med.* **30**, 119–134.
7. Cattaneo, M., Pareti, F.I., Zighetti, M., Lecchi, A., Lombardi, R., and Mannucci, P.M. (1995) Platelet aggregation at high shear is impaired in patients with congenital defects of platelet secretion and is corrected by DDAVP: correlation with the bleeding time. *J. Lab. Clin. Med.* **125**, 540–547.
8. Cattaneo, M., Lecchi, A., Randi, A.M., McGregor, J.L., and Mannucci, P.M. (1992) Identification of a new congenital defect of platelet aggregation characterized by severe impairment of platelet responses to adenosine 5'-diphosphate. *Blood* **80**, 2787–2796.
9. Nurden, P., Savi, P., Heilmann, E., Bihour, C., Herbert, J.-M., Maffrand, J.-P., and Nurden, A. (1995) An inherited bleeding disorder linked to a defective interaction between ADP and its receptor on platelets. *J. Clin. Invest.* **95**, 1612–1622.
10. Fredholm, B.B., Abbracchio, M.P., Burnstock, G., Dubyak, G.R., Harden, T.K., Jacobson, K.A., Schwabe, U., and Williams, M. (1997) Towards a revised nomenclature for P1 and P2 receptors. *Trends Pharmacol. Sci.* **18**, 79–82.
11. Gordon, J. (1986) Extracellular ATP: effects, sources and fates. *Biochem. J.* **233**, 309–319.
12. Mills, D.C.B. (1996) ADP receptor in platelets. *Thromb. Haemost.* **76**, 835–856.
13. Hourani, S.M.O. and Hall, D.A. (1994) Receptors for ADP on human platelets. *Trends Pharmacol. Sci.* **15**, 103–108.
14. Gachet, C., Hechler, B., Leon, C., Vial, C., Leray, C., Ohlmann, P., and Cazenave, J.P. (1997) Activation of ADP receptors and platelet function. *Thromb. Haemost.* **77**, 271–275.
15. Kunapuli, S.P. (1998) Functional characterization of platelet ADP receptors. *Platelets* **9**, 343–351.

16. Sage, S.O. and Rink, T. (1986) Kinetic differences between thrombin-induced and ADP-induced calcium influx and release from internal stores in fura-2-loaded human platelets. *Biochem. Biophys. Res. Commun.* **136**, 1124–1129.
17. MacKenzie, A.B., Mahaut-Smith, M.P., and Sage, S.O. (1996) Activation of receptor-operated cation channels via $P_{2X1}$ not $P_{2T}$ purinoceptors in human platelets. *J. Biol. Chem.* **271**, 2879–2881.
18. Hallam, T.J. and Rink, T.J. (1985) Response to adenosine diphosphate in human platelets loaded with the fluorescent calcium indicator Quin 2. *J. Physiol.* **368**, 131–146.
19. Daniel, J.L., Dangelmaier, C.A., Selak, M., and Smith, J.B. (1986) ADP stimulates $IP_3$ formation in human platelets. *FEBS Lett.* **206**, 299–303.
20. Daniel, J.L., Dangelmaier, C., Jin, J., Ashby, B., Smith, J.B., and Kunapuli, S.P. (1998) Molecular basis for ADP-induced platelet activation. I. Evidence for three distinct ADP receptors on platelets. *J. Biol. Chem.* **273**, 2024–2029.
21. Ohlmann, P., Laugwitz, K.L., Nuernberg, B., Spicher, K., Schultz, G., Cazenave, J.-P., and Gachet, C. (1995) The human platelet ADP receptor activates $G_{i2}$ proteins. *Biochem. J.* **312**, 775–779.
22. Cooper, D.M.F. and Rodbell, M. (1979) ADP is a potent inhibitor of human platelet plasma membrane adenylate cyclase. *Nature* **282**, 517–518.
23. Macfarlane, D.E., Gardner, S., Lipson, C., and Mills, D.C.B. (1977) Malondialdehyde production by platelets during secondary aggregation. *Thromb. Haemost.* **38**, 1002–1009.
24. Born, G.V.R. (1962) Aggregation of blood platelets by adenosine diphosphate and its reversal. *Nature* **194**, 927–929.
25. Pengo, V., Boschello, A., Marzari, A., Baca, M., Schivazappa, L., and Dalla Volta, S. (1986) Adenosine diphosphate (ADP)-induced α-granules release from platelets of native whole blood is reduced by ticlopidine but not by aspirin or dipyridamole. *Thromb. Haemost.* **56**, 147–150.
26. Rinder, C.S., Student, L.A., Bonan, J.L., Rinder, H.M., and Smith, B.R. (1993) Aspirin does not inhibit adenosine diphosphate-induced plateletα-granule release. *Blood* **82**, 505–512.
27. Rand, M.L., Perry, D.W., Packham, M.A., Gemmell, C.H., Yeo, E.L., and Kinlough-Rathbone, R.L. (1996) Conditions influencing release of granule contents from human platelets in citrated plasma induced by ADP or the thrombin receptor activating peptide SFLLRN. Direct measurement of percent release of β-thromboglobulin and assessment by flow cytometry of P-selectin expression. *Am. J. Hematol.* **52**, 288–294.
28. Kaplan, K.L., Broekman, M.J., Chernoff, A., Lesznik, G.R., and Drillings, M. (1979) Platelet α-granule proteins: Studies on release and subcellular localization. *Blood* **53**, 604–618.
29. Fagura, M.S., Dainty, I.A., McKay, G.D., Kirk, I.P., Humphries, R.G., Robertson, M.J., Dougall, I.G., and Leff, P. (1998) P2y(1)-receptors in human platelets which are pharmacologically distinct from P2y(ADP)-receptors. *Br. J. Pharmacol.* **124**, 157–164.
30. Geiger, J., Honigliedl, P., Schanzenbacher, P., and Walter, U. (1998) Ligand specificity and ticlopidine effects distinguish three human platelet ADP receptors. *Eur. J. Pharmacol.* **351**, 235–246.
31. Hechler, B., Leon, C., Vial, C., Vigne, P., Frelin, C., Cazenave, J.P., and Gachet, C. (1998) The P2y(1) receptor is necessary for adenosine 5'-diphosphate-induced platelet aggregation. *Blood* **92**, 152–159.
32. Jantzen, H.-M., Gousset, L., Bhaskar, V., Vincent, D., Tai, A., Reynolds, E., and Conley, P.D. (1999) Evidence for two distinct G protein-coupled ADP receptors mediating platelet activation. *Thromb. Haemost.* **81**, 111–117.
33. Fabre, J.E., Nguyen, M., Latour, A., Keifer, J.A., Audoly, L.P., Coffman, T.M., and Koller, B.H. (1999) Decreased platelet aggregation, increased bleeding time and resistance to thromboembolism in P2Y1-deficient mice. *Nat. Med.* **5**, 1199–1202.
34. Cattaneo, M. and Gachet, C. (1999) ADP receptors and clinical bleeding disorders. *Arterioscler. Thromb. Vasc. Biol.* **19**, 2281–2285.
35. Boyer, J.L., Romeroavila, T., Schachter, J.B., and Harden, T.K. (1996) Identification of competitive antagonists of the P2y(1) receptor. *Mol. Pharmacol.* **50**, 1323–1329.
36. Jin, J., Daniel, J.L., and Kunapuli, S.P. (1998) Molecular basis for ADP-induced platelet activation. II. The P2Y1 receptor mediates ADP-induced intracellular calcium mobilization and shape change in platelets. *J. Biol. Chem.* **273**, 2030–2034.
37. Takasaki, J., Kamohara, M., Saito, T., Matsumoto, M., Matsumoto, S., Ohishi, T., Soga, T., Matsushime, H., and Furuichi, K. (2001) Molecular cloning of the platelet P2T(AC) ADP receptor: pharmacological comparison with another ADP receptor, the P2Y(1) receptor. *Mol. Pharmacol.* **60**, 432–439.
38. Savi, P., Labouret, C., Delesque, N., Guette, F., Lupker, J., and Herbert, J.M. (2001) P2y(12), a new platelet ADP receptor, target of clopidogrel. *Biochem. Biophys. Res. Commun.* **283**, 379–383.
39. Zhang, F.L., Luo, L., Gustafson, E., Lachowicz, J., Smith, M., Qiao, X., Liu, Y.H., Chen, G., Pramanik, B., Laz, T.M., Palmer, K., Bayne, M., and Monsma, F. (2001) ADP is the cognate ligand for the orphan G-protein coupled receptor SP1999. *J. Biol. Chem.* **276**, 8608–8615.
40. Hollopeter, J., Jantzen, H.-M., Vincent, D., Li, G., England, L., Ramakrishnan, V., Yang, R.-B., Nurden, P., Nurden, A., Julius, D.J., and Conley, P.B. (2001) Identification of the platelet ADP receptor targeted by antithrombotic drugs. *Nature* **409**, 202–207.

41. Leon, C., Hechler, B., Freund, M., Eckly, A., Vial, C., Ohlmann, P., Dierich, A., LeMeur, M., Cazenave, J.P., and Gachet, C. (1999) Defective platelet aggregation and increased resistance to thrombosis in purinergic P2Y(1) receptor-null mice [see comments]. *J. Clin. Invest.* **104**, 1731–1737.
42. Foster, C.J., Prosser, D.M., Agans, J.M., Zhai, Y., Smith, M.D., Lachowicz, J.E., Zhang, F.L., Gustafson, E., Monsma, F.J., Jr., Wiekowski, M.T., Abbondanzo, S.J., Cook, D.N., Bayne, M.L., Lira, S.A., and Chintala, M.S. (2001) Molecular identification and characterization of the platelet ADP receptor targeted by thienopyridine antithrombotic drugs. *J. Clin. Invest.* **107**, 1591–1598.
43. Mahaut-Smith, M.P., Ennion, S.J., Rolf, M.G., and Evans, R.J. (2000) ADP is not an agonist at P2X(1) receptors: evidence for separate receptors stimulated by ATP and ADP on human platelets. *Br. J. Pharmacol.* **131**, 108–114.
44. Jacobson, K.A., Kim, Y.C., Wildman, S.S., Mohanram, A., Harden, T.K., Boyer, J.L., King, B.F., and Burnstock, G. (1998) A pyridoxine cyclic phosphate and its 6-azoaryl derivative selectively potentiate and antagonize activation of P2X1 receptors. *J. Med. Chem.* **41**, 2201–2206.
45. Moro, S., Guo, D., Camaioni, E., Boyer, J.L., Harden, T.K., and Jacobson, K.A. (1998) Human P2Y1 receptor: molecular modeling and site-directed mutagenesis as tools to identify agonist and antagonist recognition sites. *J. Med. Chem.* **41**, 1456–1466.
46. von Kugelgen, I. and Wetter, A. (2000) Molecular pharmacology of P2Y-receptors. *Naunyn Schmiedebergs Arch. Pharmacol.* **362**, 310–323.
47. Humphries, R.G., Robertson, M.J., and Leff, P. (1995) A novel series of $P_{2T}$ purinoceptor antagonists: definition of the role of ADP in arterial thrombosis. *Trends Pharmacol. Sci.* **16**, 179–181.
48. Jin, J., Tomlinson, W., Kirk, I.P., Kim, Y.B., Humphries, R.G., and Kunapuli, S.P. (2001) The C6-2B glioma cell P2Y(AC) receptor is pharmacologically and molecularly identical to the platelet P2Y(12) receptor. *Br. J. Pharmacol.* **133**, 521–528.
49. Mills, D.C.B., Puri, R.N., Hu, C.-J., Minnitti, C., Grana, G., Freedman, M., Colman, R.F., and Colman, R.W. (1992) Clopidogrel inhibits the binding of ADP analogues to the receptor mediating inhibition of platelet adenylate cyclase. *Atheroscler. Thromb.* **12**, 430–436.
50. Gachet, C., Cazenave, J.-P., Ohlmann, P., Bouloux, C., Defreyn, G., Driot, F., and Maffrand, J.-P. (1990) The thienopyridine ticlopidine selectively prevents the inhibitory effects of ADP but not of adrenaline on cAMP levels raised by stimulation of the adenylate cyclase of human platelets by $PGE_1$. *Biochem. Pharmacol.* **40**, 2683–2687.
51. Defreyn, G., Gachet, G., Savi, P., Driot, F., Cazenave, J.-P., and Maffrand J.-P. (1991) Ticlopidine and clopidogrel (SR25990C) selectively neutralize ADP inhibition of $PGE_1$-activated platelet adenylate cyclase in rats and rabbits. *Thromb. Haemostas.* **65**, 186–190.
52. Hechler, B., Eckly, A., Ohlmann, P., Cazenave, J.P., and Gachet, C. (1998) The P2Y1 receptor, necessary but not sufficient to support full ADP- induced platelet aggregation, is not the target of the drug clopidogrel. *Br. J. Haematol.* **103**, 858–866.
53. Savi, P., Pereillo, J.M., Uzabiaga, M.F., Combalbert, J., Picard, C., Maffrand, J.P., Pascal, M., and Herbert, J.M. (2000) Identification and biological activity of the active metabolite of clopidogrel. *Thromb. Haemost.* **84**, 891–896.
54. Vigne, P., B. Hechler, Gachet, C., Breittmayer, J.P., and Frelin, C. (1999) Benzoyl ATP is an antagonist of rat and human P2Y1 receptors and of platelet aggregation. *Biochem. Biophys. Res. Commun.* **256**, 94–97.
55. Schachter, J.B., Li, Q., Boyer, J.L., Nicholas, R.A., and Harden, T.K. (1996) Second messenger cascade specificity and pharmacological selectivity of the human P2y1-purinoceptor. *Br. J. Pharmacol.* **118**, 167–173.
56. Offermanns, S., Toombs, C.F., Hu, Y.-H., and Simon, M.I. (1997) Defective platelet activation in Gαq-deficient mice. *Nature* **389**, 183–186.
57. Paul, B.Z.S., Daniel, J.L., and Kunapuli, S.P. (1999) Platelet shape change is mediated by both calcium-dependent and -independent signaling pathways. Role of p160ROCK in platelet shape change. *J. Biol. Chem.* **274**, 28293–28300.
58. Kunapuli, S.P. (1999) Molecular events in ADP-induced platelet shape change. *Drug News Perspect.* **12**, 524–528.
59. Bauer, M., Retzer, M., Wilde, J.I., Maschberger, P., Essler, M., Aepfelbacher, M., Watson, S.P., and Siess, W. (1999) Dichotomous regulation of myosin phosphorylation and shape change by Rho-kinase and calcium in intact human platelets. *Blood* **94**, 1665–1672.
60. Sage, S.O., Yamoah, E.H., and Heemskerk, J.W. (2000) The roles of P(2X1) and P(2T AC) receptors in ADP-evoked calcium signalling in human platelets. *Cell Calcium* **28**, 119–126.
61. Rolf, M.G., Brearley, C.A., and Mahaut-Smith, M.P. (2001) Platelet shape change evoked by selective activation of P2X1 purinoceptors with alpha,beta-methylene ATP. *Thromb. Haemost.* **85**, 303–308.
62. Cusack, N.J. and Hourani, S.M.O. (1982) Adenosine diphosphate antagonists and human platelets: no evidence that aggregation and inhibition of adenylate cyclase are mediated by different receptors. *Br. J. Pharmacol.* **76**, 221–227.
63. Macfarlane, D.E. and Mills, D.C.B. (1975) The effects of ATP on platelets. Evidence against the central role of ADP in primary aggregation. *Blood* **46**, 309–320.

64. Jin, J., and Kunapuli, S.P. (1998) Co-activation of two different G protein-coupled receptors is essential for ADP-induced platelet aggregation. *Proc. Natl. Acad. Sci. U. S. A.* **95**, 8070–8074.
65. Kim, Y.B., Jin, J., Dangelmaier, C., Daniel, J.L., and Kunapuli, S.P. (1999) The P2Y1 receptor is essential for ADP-induced platelet shape change and aggregation in mouse platelets. *Platelets* **10**, 399–406.
66. Yang, J., Wu, J., Kowalska, M.A., Dalvi, A., Prevost, N., O'Brien, P.J., Manning, D., Poncz, M., Lucki, I., Blendy, J.A., and Brass, L.F. (2000) Loss of signaling through the G protein, Gz, results in abnormal platelet activation and altered responses to psychoactive drugs. *Proc. Natl. Acad. Sci. U. S. A.* **97**, 9984–9989.
67. Savi, P., Beauverger, P., Labouret, C., Delfaud, M., Salel, V., Kaghad, M., and Herbert, J.M. (1998) Role of P2Y1 purinoceptor in ADP-induced platelet activation. *FEBS Lett.* **422**, 291–295.
68. Paul, B.Z.S., Jin, J., and Kunapuli, S.P. (1999) Molecular mechanism of thromboxane A2- induced platelet aggregation: Essential role for P2T*AC* and α2A receptors. *J. Biol. Chem.* **274**, 29108–29114.
69. Ishii-Watabe, A., Uchida, E., Mizuguchi, H., and Hayakawa, T. (2000) On the mechanism of plasmin-induced platelet aggregation. Implications of the dual role of granule ADP. *Biochem. Pharmacol.* **59**, 1345–1355.
70. Savi, P., Bornia, J., Salel, V., Delfaud, M., and Herbert, J.M. (1997) Characterization of P2x1 purinoreceptors on rat platelets - Effect of clopidogrel. *Br. J. Haematol.* **98**, 880–886.
71. Jin, J., Quinton, T., Zhang, J., Rittenhouse, S., and Kunapuli, S.P. (2002) ADP-induced thromboxane A2 generation in human platelets requires coordinated signaling throough integrin αIIBβ3 and ADP receptors. *Blood* **99**, 193–198.
72. Dangelmaier, C., Jin, J., Smith, J.B., and Kunapuli, S.P. (2001) Potentiation of thromboxane A2-induced platelet secretion by Gi signaling through the phosphoinositide-3 kinase pathway. *Thromb. Haemost.* **85**, 341–348.
73. Storey, R.F., Sanderson, H.M., White, A.E., May, J.A., Cameron, K.E., and Heptinstall, S. (2000) The central role of the P(2T) receptor in amplification of human platelet activation, aggregation, secretion and procoagulant activity. *Br. J. Haematol.* **110**, 925–934.
74. Cattaneo, M., Lecchi, A., Lombardi, R., Gachet, C., and Zighetti, M.L. (2000) Platelets from a patient heterozygous for the defect of P2CYC receptors for ADP have a secretion defect despite normal thromboxane A2 production and normal granule stores: further evidence that some cases of platelet 'primary secretion defect' are heterozygous for a defect of P2CYC receptors. *Arterioscler. Thromb. Vasc. Biol.* **20**, E101–106.
